# Patient subgroup analyses of the treatment effect of subcutaneous interferon β-1a on development of multiple sclerosis in the randomized controlled REFLEX study

**DOI:** 10.1007/s00415-013-7222-6

**Published:** 2014-01-12

**Authors:** Mark S. Freedman, Nicola De Stefano, Frederik Barkhof, Chris H. Polman, Giancarlo Comi, Bernard M. J. Uitdehaag, Florence Casset-Semanaz, Brian Hennessy, Lorenz Lehr, Bettina Stubinski, Dominic L. Jack, Ludwig Kappos

**Affiliations:** 1 Department of Medicine, University of Ottawa, 501 Smyth Road, Ottawa, ON K1H 8L6 Canada; 2 Department of Neurological and Behavioral Sciences, University of Siena, Siena, Italy; 3 Diagnostic Radiology, VU University Medical Center, Amsterdam, The Netherlands; 4 Department of Neurology, VU University Medical Center, Amsterdam, The Netherlands; 5 Department of Neurology and Institute of Experimental Neurology, University Vita-Salute IRCCS, San Raffaele Hospital, Milan, Italy; 6 EMD Serono, Inc., Billerica, MA USA; 7 Merck Serono S.A., Geneva, Switzerland; 8 Caudex Medical, Oxford, UK; 9 Departments of Neurology and Biomedicine, University Hospital Basel, Basel, Switzerland

**Keywords:** Interferon beta, First clinical demyelinating event, Clinically isolated syndrome, McDonald MS, Clinically definite MS

## Abstract

The REFLEX study (NCT00404352) established that subcutaneous (sc) interferon (IFN) β-1a reduced the risks of McDonald MS (2005 criteria) and clinically definite multiple sclerosis (CDMS) in patients with a first clinical demyelinating event suggestive of MS. The aim of this subgroup analysis was to assess the treatment effect of sc IFN β-1a in patient subgroups defined by baseline disease and demographic characteristics (age, sex, use of steroids at the first event, classification of first event as mono- or multifocal, presence/absence of gadolinium-enhancing lesions, count of <9 or ≥9 T2 lesions), and by diagnosis of MS using the revised McDonald 2010 MS criteria. Patients were randomized to the serum-free formulation of IFN β-1a, 44 μg sc three times weekly or once weekly, or placebo, for 24 months or until diagnosis of CDMS. Treatment effects of sc IFN β-1a on McDonald 2005 MS and CDMS in the predefined subgroups were similar to effects found in the intent-to-treat population. McDonald 2010 MS was retrospectively diagnosed in 37.7 % of patients at baseline. Both regimens of sc IFN β-1a significantly reduced the risk versus placebo of McDonald 2005 MS and CDMS, irrespective of McDonald 2010 status at baseline (risk reductions between 29 and 51 %). The effect of sc IFN β-1a was not substantially influenced by baseline patient demographic and disease characteristics, or baseline presence/absence of McDonald 2010 MS.

## Introduction

The first presentation of relapsing multiple sclerosis (MS) is the occurrence of an acute first clinical demyelinating event. The risk of new clinical and/or magnetic resonance imaging (MRI) events can be determined by a number of findings, such as the presence of specific oligoclonal bands in the cerebrospinal fluid, subclinical abnormalities in visual evoked potentials, the number of T2 lesions, the number of gadolinium-enhancing (Gd+) lesions and the type of clinical presentation (monofocal or multifocal) [1–7]. Occasionally, lesions within the central nervous system (CNS) characteristic of MS may be detected by MRI in the absence of clinical symptoms (radiologically isolated syndrome) [8].

The McDonald criteria for the diagnosis of MS have been continually reviewed and updated as necessary since their initial publication in 2001 [9, 10]. The most recent version of the criteria (McDonald 2010 [11]) permits the diagnosis of MS at the time of the first clinical demyelinating event without a requirement for a second attack, provided certain MRI findings are met. This means that some patients who would not have been diagnosed as having MS at the time of the first attack (clinically isolated syndrome) using the 2005 criteria would now be diagnosed as having MS on the basis of having both one or more Gd+ lesions (not related to the attack) and non-enhancing lesions on MRI.

The REFLEX (REbif FLEXible dosing in early MS; clinicaltrials.gov identifier NCT00404352) trial demonstrated that, in patients with a first clinical demyelinating event at high risk of conversion to MS, interferon (IFN) β-1a, 44 μg subcutaneously (sc) three times weekly (tiw) or once weekly (qw), significantly delayed conversion to McDonald MS (defined by the 2005 criteria) and clinically definite MS (CDMS), and significantly reduced MRI measures of disease activity versus placebo over 24 months [12]. The efficacy of tiw dosing was significantly greater than that of qw dosing for conversion to McDonald MS and MRI outcomes [12].

This paper will report the result of analyses of prespecified patient subgroups in the REFLEX trial. The patient subgroups included those factors used to balance the population during randomization and others that have previously been identified as risk factors for McDonald MS or CDMS. With the revision to the McDonald guidelines [11], it was also important to establish whether sc IFN β-1a was effective in the subgroup of patients who would still have been classified as not having MS by the updated criteria, and also to monitor the time to the second clinical event in patients who would have been diagnosed as already having MS at the time of their inclusion in the REFLEX study.

## Methods

The full methodology of this trial has been published previously [12]. Briefly, patients with a first clinical demyelinating event suggestive of MS were randomized 1:1:1 to the serum-free formulation of IFN β-1a, 44 μg sc tiw or qw, or placebo, for 24 months or until a diagnosis of CDMS. An independent adjudication committee was responsible for assessing the eligibility of all patients, confirming or reclassifying each patient’s first event as mono- or multifocal and reviewing all relapses that contributed to a diagnosis of CDMS. This committee was blinded to treatment group and comprised neurologists and a neuroradiologist with expertise in MS. The reporting and evaluation of relapses were continuous and independent of scheduled visits. A relapse was defined as new or worsening neurological symptoms, in the absence of fever, lasting for at least 24 h, accompanied by an objective change in the relevant (i.e., symptomatic) functional system, and preceded by 30 days or more of clinical stability or improvement. MRI scans were evaluated centrally at the VU Medical Center, Amsterdam, the Netherlands. The study was undertaken in compliance with the Declaration of Helsinki and standards of Good Clinical Practice according to the International Conference on Harmonisation of Technical Requirements for Registration of Pharmaceuticals for Human Use. Before initiation of the trial at each centre, the relevant institutional review board or independent ethics committee reviewed and approved the trial protocol, patient information leaflet, informed consent forms, and investigator brochure.

The predefined subgroups in this analysis were those used to stratify the randomization process: age <30 or ≥30 years, use of steroids at first attack, multi- or monofocal presentation of first clinical demyelinating event, presence or absence of Gd+ lesions at baseline. Other predefined subgroups were sex and the presence of <9 or ≥9 T2 lesions at baseline.

In a post hoc analysis, a simulated retrospective diagnosis at baseline of dissemination in time and space according to the McDonald 2010 MS criteria was conducted for each patient. Evidence of dissemination in time was defined as the simultaneous presence of Gd+ and non-enhancing lesions (with the exception of patients with brainstem syndromes). Dissemination in space was defined as the presence of ≥1 T2 lesions in ≥2 of three MS-typical regions of the CNS; in the original REFLEX MRI analysis, spinal cord location was not assessed and, therefore, only the juxtacortical, periventricular, and infratentorial regions were analyzed [12]. Patients were divided into McDonald 2010-positive and -negative subgroups.

All subgroup analyses were considered to be exploratory.

### Statistical analyses

The primary endpoint of the REFLEX study was time to conversion to McDonald MS as defined by the 2005 version of the criteria [10]. The main secondary endpoint was time to conversion to CDMS. As described previously [12], the probability of patients remaining event free over time (from randomization) in each of the three treatment groups was estimated using the nonparametric Kaplan–Meier method for the primary and main secondary endpoints. The hazard ratios (HRs) for between-group comparisons with corresponding two-sided 95 % confidence intervals (CIs) in the prespecified subgroups were estimated using an unadjusted univariate Cox’s proportional hazard model with treatment as the only covariate. *p* values for the comparison between treatment groups within each subgroup were calculated using the same model. Treatment effects within McDonald 2010 MS status strata were analyzed post hoc using Cox’s proportional hazard regression models, with treatment as a covariate.

## Results

The intent-to-treat (ITT) population comprised all 517 patients randomized to treatment. As previously described, baseline demographic and disease characteristics were similar across treatment groups [12]. Baseline characteristics of the ITT population, stratified by retrospective McDonald 2010 status, are summarized in Table 1.

**Table 1:** Baseline demographic, disease and MRI characteristics

Characteristic	Intent-to-treat (*n* = 517)	McDonald 2010 MS-negative (*n* = 322)	McDonald 2010 MS-positive (*n* = 195)
Age, years, mean (SD)	30.7 (8.2)	31.8 (8.3)	28.9 (7.6)
Women, *n* (%)	332 (64.2)	205 (63.7)	127 (65.1)
Time since first demyelinating event, days, mean (SD)	57.6 (3.8)	57.7 (3.7)	57.5 (4.0)
Classification of first clinical demyelinating event as monofocal^a^, *n* (%)	277 (53.6)	192 (59.6)	85 (43.6)
Steroid use at first clinical demyelinating event, *n* (%)	365 (70.6)	239 (74.2)	126 (64.6)
Number of T1 Gd+ lesions, mean (SD)	1.3 (2.9)	0.1 (0.8)^b^	3.3 (3.9)
Presence of Gd+ lesions at baseline, *n* (%)	213 (41.2)	18 (5.6)^b^	195 (100)
Number of T2 lesions, mean (SD)	22.3 (20.0)	17.6 (15.7)^b^	29.8 (23.4)
Presence of ≥9 T2 lesions at baseline, *n* (%)	377 (72.9)	201 (63.0)^b^	174 (89.2)

*Gd*+ gadolinium-enhancing, *MRI* magnetic resonance imaging, *MS* multiple sclerosis, *SD* standard deviation

^a^According to the adjudication committee

^b^ *n* = 319

### Conversion to MS in subgroup populations

#### McDonald MS 2005

**Fig. 1:**
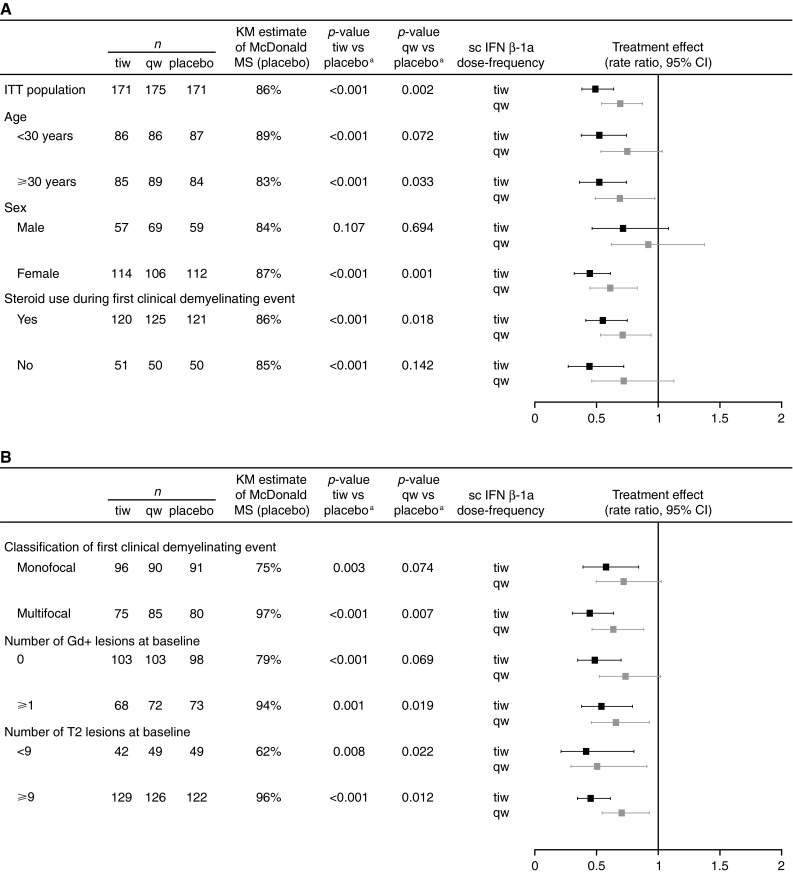
Analysis of the time to McDonald 2005 MS according to prespecified subgroups of **a** patient demographics and steroid treatment, and **b** MRI findings at the first clinical demyelinating event (ITT population; *n* = 517). *Footnote a* *p* value calculated by a multivariate Cox proportional hazards model. *CI* confidence interval, *Gd*+ gadolinium-enhancing, *IFN* interferon, *ITT* intent-to-treat, *KM* Kaplan–Meier, *MRI* magnetic resonance imaging, *MS* multiple sclerosis, *qw* once weekly, *sc* subcutaneous, *tiw* three times weekly

Subgroups defined by patient demographics (age <30 vs. ≥30 years), sex (female vs male), and steroid use during the first clinical demyelinating event (yes vs. no) did not increase the risk of McDonald 2005 MS (*p* > 0.05 for all three covariates). This is reflected in the proportion of placebo-treated patients with McDonald 2005 MS at 2 years that were similar (range 83–89 %; Fig. 1a) across these subgroups.

There was a significantly increased risk of McDonald 2005 MS in patient subgroups with evidence of more severe disease activity at baseline (Fig. 1b): ≥1 Gd+ lesions versus no Gd+ lesions, ≥9 T2 lesions vs. <9 T2 lesions, and in patients with multifocal versus monofocal presentation of the first clinical demyelinating event (*p* < 0.05 for all three covariates). This is reflected in the proportion of placebo-treated patients with McDonald 2005 MS at 2 years (range 62–97 %; Fig. 1b).

In general, the treatment effects of sc IFN β-1a were similar between predefined subgroups and similar to those found in the overall ITT population (Fig. 1a). A significant treatment effect of sc IFN β-1a tiw versus placebo was found in all subgroups apart from the subgroup of male patients. A significant treatment effect of sc IFN β-1a qw versus placebo was observed in the following subgroups: age ≥30 years, female patients, patients who used steroids for the first clinical event, patients with multifocal presentation, patients with ≥1 Gd+ lesion at baseline, and patients with <9 and ≥9 T2 lesions at baseline (Fig. 1a, b).

#### CDMS

**Fig. 2:**
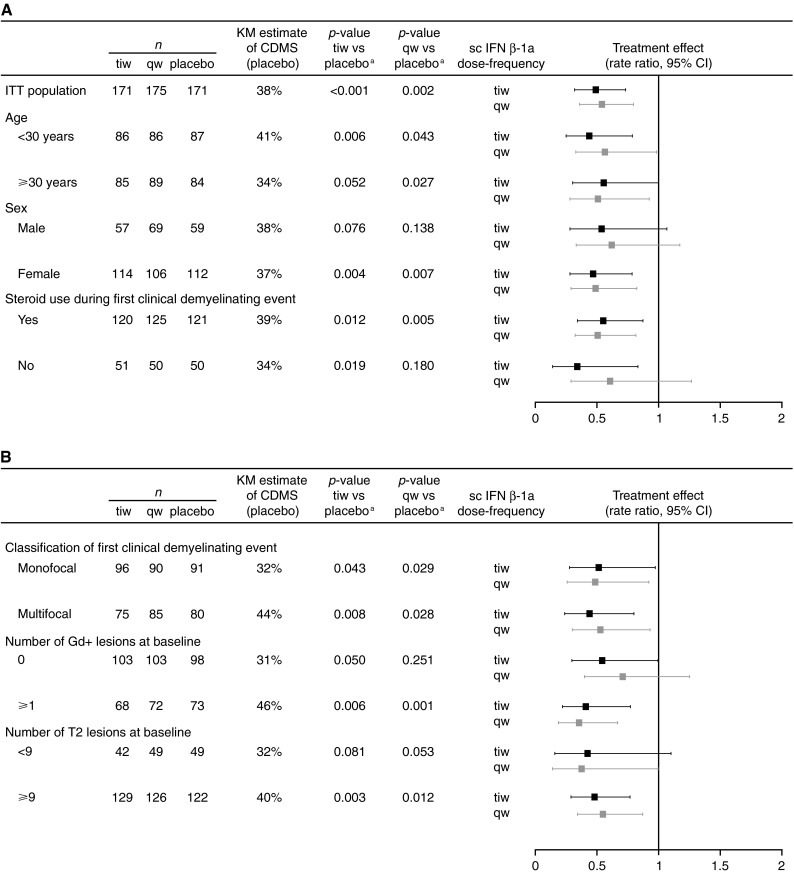
Analysis of the time to CDMS according to prespecified subgroups of **a** patient demographics and steroid treatment, and **b** MRI findings at the first clinical demyelinating event (ITT population; *n* = 517). *Footnote a* *p* value calculated by a multivariate Cox proportional hazards model. *CI* confidence interval, *CDMS* clinically definite multiple sclerosis, *Gd*+ gadolinium-enhancing, *IFN* interferon, *ITT* intent-to-treat, *KM* Kaplan–Meier, *MRI* magnetic resonance imaging, *qw* once weekly, *sc* subcutaneous, *tiw* three times weekly

In subgroups defined by patient demographics and steroid treatment, estimates of CDMS in placebo-treated patients at 2 years were in the range of 34–41 % (Fig. 2a). In subgroups defined by MRI findings at baseline, estimates of CDMS at 2 years were in the range of 31–46 % in placebo-treated patients (Fig. 2b). None of the predefined baseline subgroups examined showed an effect on time to CDMS in the Cox proportional hazards regression model (*p* > 0.05 in all cases).

As previously reported [12], time to CDMS compared with placebo was significantly reduced by both dose-frequencies of sc IFN β-1a, with no significant difference between tiw and qw dosing. The treatment effects of sc IFN β-1a in the predefined subgroups were similar to those in the ITT population (Fig. 2).

### Post hoc analysis of McDonald 2010 MS status at baseline

Retrospective application of the McDonald 2010 criteria found that 195/517 (37.7 %) of patients in the ITT population in REFLEX would have been classified as having had MS at baseline (Table 2).

**Table 2:** Baseline characteristics leading to retrospective McDonald 2010 MS diagnosis

	IFN β-1a, 44 μg sc tiw (*n* = 171)	IFN β-1a, 44 μg sc qw (*n* = 175)	Placebo (*n* = 171)	Overall (*n* = 517)
≥1 Gd+ lesions at baseline and number of T2 lesions > number of Gd+ lesions	68 (39.8)	72 (41.1)	73 (42.7)^a^	212 (41.0)
≥1 T2 lesions in two of three locations (periventricular, juxtacortical or infratentorial)^b^ and/or multifocal presentation (by adjudication committee)	144 (84.2)	148 (84.6)	139 (81.3)	431 (83.4)
Fulfilling McDonald 2010 MS criteria^c^ (both of the above)	62 (36.3)	66 (37.7)	67 (39.2)	195 (37.7)

Data are presented as *n* (%)

*Gd*+ gadolinium-enhancing, *IFN* interferon, *MS* multiple sclerosis, *qw* once weekly, *sc* subcutaneously, *SD* standard deviation, *tiw* three times weekly

^a^One patient was excluded from the McDonald 2010-positive population because an additional criterion of having a greater number of T2 lesions than Gd+ lesions at baseline was not met

^b^According to the investigator

^c^The McDonald 2010 MS criteria specify ≥1 T2 lesions in ≥2 of four MS-typical regions of the central nervous system; the REFLEX study did not assess spinal cord location

Baseline characteristics of patients with retrospective McDonald 2010 diagnosis were generally similar to those of the ITT population, with the expected exceptions of a higher mean number of Gd+ and T2 lesions and a lower proportion of patients with monofocal presentation (Table 1).

#### Effect on risk of McDonald 2005 MS

**Fig. 3:**
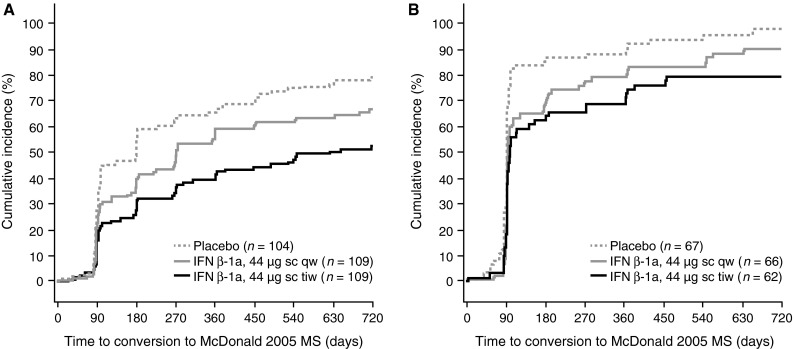
Kaplan–Meier cumulative incidence curves for time to conversion to McDonald 2005 MS in patients negative (**a**) and positive (**b**) for McDonald 2010 MS at baseline. *IFN* interferon, *MS* multiple sclerosis, *qw* once weekly, *sc* subcutaneously, *tiw* three times weekly

In placebo-treated patients, the risk of McDonald 2005 MS at 2 years was 79 % in patients who were McDonald 2010 MS-negative at baseline (Fig. 3a) and 97 % in patients who were McDonald 2010 MS-positive at baseline (Fig. 3b), compared with 86 % in all patients. The risk of McDonald 2005 MS was significantly higher in patients who were McDonald 2010 MS-positive compared with McDonald 2010 MS-negative at baseline (covariate effect for McDonald 2010 MS-positive vs. -negative at baseline; HR 2.25, 95 % CI 1.83–2.77; *p* < 0.001).

**Fig. 4:**
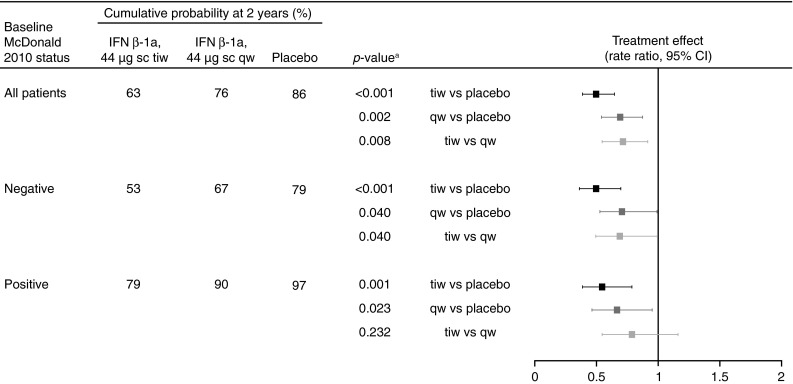
Conversion to McDonald 2005 MS, by McDonald 2010 MS status at baseline. *Footnote a* *p* value calculated by a Cox proportional hazards model, with treatment and MS status (McDonald or clinically definite MS: yes or no) as covariates. *CI* confidence interval, *IFN* interferon, *MS* multiple sclerosis, *qw* once weekly, *sc* subcutaneously, *tiw* three times weekly

The treatment effect of sc IFN β-1a versus placebo on McDonald 2005 MS was significant in patients who were positive (risk reductions: tiw, 46 %; qw, 34 %) and negative (risk reductions: tiw, 51 %; qw, 29 %) for McDonald 2010 MS at baseline (Fig. 4). There was a significantly greater treatment effect of tiw versus qw in patients negative for McDonald 2010 MS at baseline, with a similar but non-significant trend in patients positive for McDonald 2010 MS.

#### Effect on risk of CDMS

**Fig. 5:**
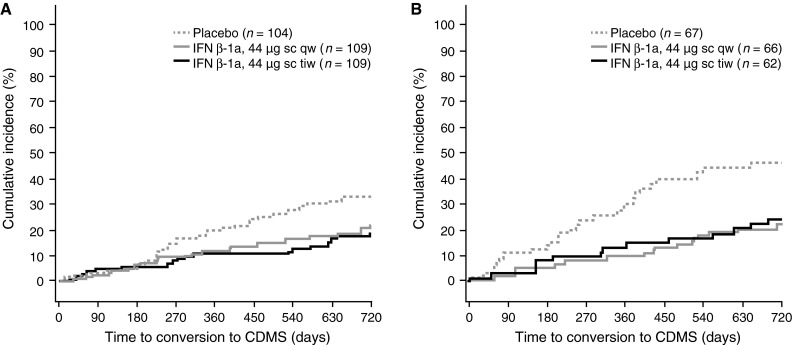
Kaplan–Meier cumulative incidence curves for time to conversion to CDMS in patients negative (**a**) and positive (**b**) for McDonald 2010 MS at baseline. *CDMS* clinically definite multiple sclerosis, *IFN* interferon, *qw* once weekly, *sc* subcutaneously, *tiw* three times weekly

There was a borderline, non-significant increased risk of developing CDMS in McDonald 2010-positive versus -negative patients (covariate effect for McDonald 2010 MS-positive vs. -negative at baseline; HR 1.38, 95 % CI 0.97–1.95; *p* = 0.0704). This is reflected in placebo-treated patients, in whom the risk of CDMS at 2 years was 32 % in those McDonald 2010 MS-negative (Fig. 5a) and 46 % in those McDonald 2010 MS-positive (Fig. 5b) at baseline (corresponding proportions at 1 year: McDonald 2010-negative 19 %, McDonald 2010-positive 29 %).

**Fig. 6:**
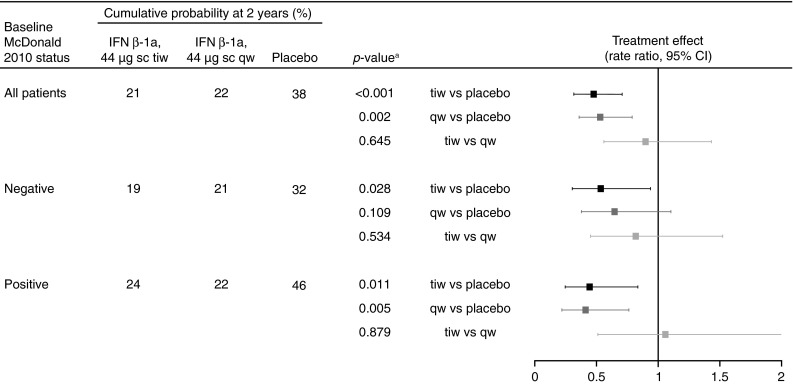
Conversion to CDMS, by McDonald 2010 MS status at baseline. *Footnote a* *p* value calculated by a Cox proportional hazards model, with treatment as a covariate. *CDMS* clinically definite multiple sclerosis, *CI* confidence interval, *IFN* interferon, *qw* once weekly, *sc* subcutaneously, *tiw* three times weekly

The treatment effect of sc IFN β-1a tiw versus placebo on time to CDMS was significant in patients positive or negative for McDonald 2010 MS at baseline (risk reductions: 56 and 47 %, respectively; Fig. 6). The treatment effect of sc IFN β-1a qw versus placebo on time to CDMS was significant in patients who were McDonald 2010 MS-positive at baseline, but not in those who were McDonald 2010 MS-negative (risk reductions: 59 and 36 %, respectively). There was no significant difference in treatment effect of tiw versus qw, irrespective of McDonald 2010 MS status at baseline.

## Discussion

Previous studies of patients with a first clinical demyelinating event suggestive of MS have found that certain patient characteristics, such as younger age, more active disease as assessed by MRI, and steroid use during the first event, are predictive of CDMS [7, 13]. In the REFLEX study, the primary outcome was McDonald 2005 MS. Multifocal presentation, the presence of Gd+ lesions and ≥9 T2 lesions at baseline predicted a higher risk of McDonald 2005 MS at 2 years. Age, sex, and steroid use during the first clinical demyelinating event did not predict a higher risk of McDonald 2005 MS at 2 years. In contrast to previous studies, none of the subgroups examined predicted a higher risk of CDMS at 2 years, although there was a trend towards an increased risk in patients with multifocal presentation and presence of Gd+ lesions and ≥9 T2 lesions at baseline, similar to that observed for conversion to McDonald 2005 MS.

The treatment effect on McDonald 2005 MS with both dose-frequencies of sc IFN β-1a in the predefined subgroups was broadly consistent with the findings of the REFLEX ITT analysis [12]. This suggests that the treatment effect of sc IFN β-1a is not dependent on demographic or disease characteristics. An interesting exception to this was the reduced treatment effect in male compared to female patients. However, the small number of male patients in each treatment group might have limited the ability to detect a significant treatment effect.

The treatment effect of sc IFN β-1a on McDonald 2005 MS was generally greater with tiw than with qw dosing, the difference reaching statistical significance in several subgroups. This is consistent with the clear additional benefit of tiw over qw dose-frequency on McDonald 2005 MS, as seen in the ITT population [12].

The McDonald criteria for the diagnosis of MS have been revised since the REFLEX study started. The changes to the criteria potentially allow the earlier diagnosis of MS such that, using a retrospective diagnosis of McDonald 2010 MS, over a third of the patients enrolled in REFLEX already had MS. An important question is whether excluding patients positive for McDonald 2010 MS at baseline would have affected the primary and main clinical secondary endpoints of the REFLEX study. In patients who were McDonald 2010 MS-positive at baseline, the endpoint of McDonald 2005 MS potentially provides a useful composite endpoint of new MRI lesions or a relapse. The baseline characteristics of patients with a retrospective McDonald 2010 diagnosis were similar to those of the ITT population, with the exception of a higher number of Gd+ lesions and T2 lesions, and a lower rate of monofocal presentation. These differences were expected as the 2010 definition of dissemination in time (simultaneous detection of Gd+ and T2 lesions) meant that all patients classified as having McDonald 2010 MS had Gd+ lesions. Similarly, the 2010 definition of dissemination in space (≥1 T2 lesions in ≥2 of four MS-typical regions of the CNS) selected for patients with higher numbers of T2 lesions and wider dissemination within the CNS [11].

Retrospective diagnosis of McDonald 2010 MS at baseline increased the risk of McDonald 2005 MS (i.e., a greater risk of new MRI findings or relapse at some point in the 2 years of the study) at 2 years in patients treated with placebo. This was expected from the predefined subgroup analyses, in which an increased risk of McDonald 2005 MS was observed in patients with Gd+ lesions or ≥9 T2 lesions at baseline. Both dose-frequencies of sc IFN β-1a showed significant treatment effects versus placebo on the risk of McDonald 2005 MS, irrespective of McDonald 2010 MS status at baseline. There was a significant difference in treatment effect between tiw and qw dosing in patients negative for McDonald 2010 MS at baseline. These results suggest that the primary finding of the REFLEX study would be the same if patients positive for McDonald 2010 MS were excluded. In other words, sc IFN β-1a can delay McDonald MS in patients with a first clinical demyelinating event, with evidence of a significantly greater effect in patients receiving sc IFN β-1a tiw versus qw. In patients who were positive for McDonald 2010 MS at baseline, there was a trend towards a difference, albeit not significant, between tiw and qw dosing in the treatment effect on McDonald 2005 MS.

McDonald 2010 MS status at baseline did not apparently affect time to CDMS in patients treated with placebo, as McDonald 2010 MS status in the post hoc analysis was not a significant covariate in the qw versus placebo model, and was of only borderline significance in the tiw versus placebo model. This might have been expected from the analysis of the predefined subgroups, as there was no significant effect of the number of Gd+ or T2 lesions at baseline on time to CDMS. The treatment effect of sc IFN β-1a on CDMS was similar to that observed in the ITT population, irrespective of McDonald 2010 MS status at baseline.

The analysis of the McDonald 2010 MS-positive population offers some insight into the clinical efficacy of sc IFN β-1a in the early phase of relapsing–remitting MS. Although the REFLEX study was not powered to detect clinical efficacy in this subgroup, both sc IFN β-1a tiw and qw showed a significant reduction versus placebo in the occurrence of McDonald 2005 MS (i.e., new MRI findings or relapse) and CDMS.

The results of these subgroup analyses of data from the REFLEX study show that the treatment effects of sc IFN β-1a at both dose frequencies across all subgroups were consistent with the treatment effects in the ITT population, and that there was a clear additional benefit of tiw over qw dose frequency for time to McDonald MS. These treatment effects were also similar across subgroups in which patients were stratified by retrospective diagnosis of McDonald MS by the 2010 criteria, suggesting that had these criteria been available at the time of study design, the main conclusions of the REFLEX study, that sc IFN β-1a reduced the risk of McDonald 2005 MS and CDMS in patients with a first clinical demyelinating event, would not have been affected. Taken together, these analyses add to the previously reported findings of the REFLEX study, in that a consistent treatment effect of both dosing frequencies is seen across all patient groups, with a more robust effect of tiw over qw dosing on McDonald 2005 MS and MRI lesion counts. This further supports the initiation of treatment with sc IFN β-1a at the time of the first clinical demyelinating event.
